# The Effect of Yacon Consumption on Glycemic Control and Lipid Profiles: A Systematic Review and Meta‐Analysis of Randomised Controlled Trials

**DOI:** 10.1002/edm2.70121

**Published:** 2025-10-28

**Authors:** Maryam Nilghaz, Fatemeh Sheikhhossein, Mahnoush Mehrzad Samarin, Mohammad Reza Amini, Mahsa Elahikhah, Moharam Jalalzadeh, Maryam Khakbaz, Negin Lohrasbi, Sajjad Etesamnia, Fatemeh Torkizadeh, Azita Hekmatdoost

**Affiliations:** ^1^ Student Research Committee, Department of Clinical Nutrition & Dietetics National Nutrition & Food Technology Research Institute, Shahid Beheshti University of Medical Sciences Tehran Iran; ^2^ Department of Clinical Nutrition, School of Nutritional Sciences and Dietetics Tehran University of Medical Sciences (TUMS) Tehran Iran; ^3^ Master of Sport Physiology, Education and Sport Science Department of Physical Education and Sport Sciences, Science and Research Branch Islamic Azad University Tehran Iran; ^4^ Nutrition and Food Security Research Center, School of Nutrition and Food Science Isfahan University of Medical Sciences Isfahan Iran; ^5^ Department of Nutrition Sciences, School of Paramedical Sciences Ahvaz Jundishapur University of Medical Sciences Ahvaz Iran; ^6^ Department of Community Nutrition, School of Nutritional Sciences and Dietetics Tehran University of Medical Sciences (TUMS) Tehran Iran; ^7^ Student Research Committee, School of Public Health Iran University of Medical Sciences Tehran Iran; ^8^ Department of Nutrition, School of Public Health Iran University of Medical Sciences Tehran Iran; ^9^ Department of Clinical Nutrition & Dietetics National Nutrition & Food Technology Research Institute, Shahid Beheshti University of Medical Sciences Tehran Iran

**Keywords:** glycemic control, lipid profile, meta‐analysis, RCTs, yacon

## Abstract

**Background:**

Recent human studies have indicated the beneficial effects of yacon on diabetes and metabolic syndrome; however, no meta‐analysis has investigated the effects of yacon on glycemic control and lipid profiles.

**Methods:**

Searches were conducted in five databases—PubMed, Web of Science, Scopus, Google Scholar, Cochrane Library—and relevant randomised controlled trials (RCTs) until June 2024. The random‐effects model was employed to compute the effect size, thereafter represented as a weighted mean difference (WMD) and a 95% confidence interval (CI). This study's registration number in PROSPERO is CRD420251028504.

**Results:**

This study integrated seven RCTs with 239 participants. The results demonstrated that yacon consumption had no statistically significant effects on fasting blood sugar (FBS, *p* = 0.33), insulin levels (*p* = 0.76), homeostasis model assessment for insulin resistance (HOMA‐IR, *p* = 0.42), total cholesterol (TC, *p* = 0.17), low‐density lipoprotein (LDL, *p* = 0.12), high‐density lipoprotein (HDL, *p* = 0.42), or triglycerides (TG, *p* = 0.75). However, subgroup studies indicated that yacon consumption reduced FBS levels over an exceeding 8‐week duration in both sexes and in persons over 40. Furthermore, yacon intake resulted in a decrease in LDL‐cholesterol levels for more than 8 weeks, particularly in women and individuals over 40. Additionally, it led to a decrease in LDL‐cholesterol levels among women and individuals over 40 who consumed yacon for more than 8 weeks, and HDL‐cholesterol levels increased in those aged 40 and above.

**Conclusion:**

Overall, this meta‐analysis indicates that yacon use in adults does not lead to significant improvements in lipid profiles or glycemic parameters.

## Graphical abstract

Yacon did not significantly affect FBS, insulin levels, HOMA‐IR, TC, LDL, HDL, and TG. Yacon consumption reduced FBS levels over an exceeding 8‐week duration in both sexes and in persons over 40.

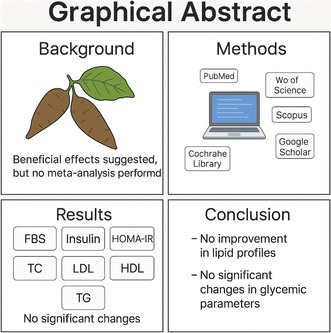

## 1 Introduction

The number of people with diabetes is expected to rise to 853 million by 2050. Global diabetes prevalence is estimated to increase by 59.7% between 2021 and 2050, reaching 9.8% by 2050 [1]. Nutritional strategies offer alternatives to pharmaceutical methods for reducing hyperglycemia and metabolic disorders [2, 3, 4]. Yacon (*Smallanthus sonchifolius*) is a perennial herbal plant that belongs to the Asteraceae family and was originally found in the Andean region of South America [5]. During the past decades, the popularity of yacon has expanded to various countries in Asia and Europe [6]. Various products derived from yacon tubers and leaves, such as syrup, powder, and herbal tea, have been developed, and their beneficial effects are demonstrated by scientific research [7]. Yacon roots are a low‐calorie natural food with plenty of fibre and a high water content [8]. They also contain fructooligosaccharides (FOS), along with bioactive substances like chlorogenic acid and various phenolic compounds [9]. These components exhibit antioxidant, prebiotic, and antimicrobial properties and are considered beneficial functional foods [10, 11].

Previous studies have indicated that yacon offers several health benefits, including enhancements in managing diabetes, metabolic syndrome, and aiding in weight management [12, 13, 14]. Yacon contains FOS, small soluble dietary fibres that promote the beneficial gut bacteria like bifidobacteria and lactobacilli, and modulate microbiota to reduce colorectal cancer risk, enhance immunity, and improve glucose absorption [15, 16, 17, 18]. Also, FOS consumption reduces inflammation and enhances insulin sensitivity through the production of short‐chain fatty acids (SCFAs) [19, 20, 21, 22]. In obese individuals, SCFAs mitigate insulin resistance by reducing plasma free fatty acid (FFA) levels [16]. Additionally, SCFAs stimulate the secretion of gut hormones, such as peptide YY (PYY) and glucagon‐like peptide‐1 (GLP‐1), which control plasma glucose levels [23, 24]. SCFAs also modulate pathways involving lipid metabolism by inhibiting lipolysis, enhancing triglyceride (TG) mobilisation, promoting adipogenic differentiation, and reducing cholesterol synthesis [25, 26, 27]. Numerous animal studies revealed that yacon and its extracts improve beta cell function, insulin resistance, plasma insulin, and glucose levels, while reducing TG and very low‐density lipoprotein (VLDL) in diabetic rats and lowering postprandial serum TG in normal rats [13, 28, 29, 30, 31]. In human studies, yacon syrup consumption in obese and slightly dyslipidemic pre‐menopausal women significantly reduced body weight, waist circumference, and body mass index, fasting serum insulin, and serum low‐density lipoprotein (LDL) levels. However, fasting glucose and serum lipids were not affected by yacon syrup consumption [13]. Another study found that consumption of yacon syrup did not affect the glycemic profile, although it decreased total cholesterol and body fat in overweight or obese women [32]. Several randomised clinical trials (RCTs) have examined the effects of yacon on glycemic profile and serum lipid levels; however, the findings were inconsistent. While some studies report beneficial effects on glucose and insulin concentrations, LDL or total cholesterol (TC) levels [12, 13, 32, 33], others found no significant effects [12, 32, 34]. Differences in yacon form (e.g., syrup vs. powder), dosage, or study populations may explain the inconsistent findings. Numerous previous reviews have investigated the prebiotics on glycaemic and lipid profiles. A recently published meta‐analysis found no significant changes in metabolic profiles among adults with prediabetes [35]. However, two systematic reviews and meta‐analyses in overweight and obese adults and diabetic individuals demonstrated beneficial effects of prebiotics and synbiotics on lipid profiles and glucose homeostasis [36, 37]. Additionally, a recent systematic review found that yacon syrup consumption was associated with improved metabolic parameters, including reduced fasting insulin, homeostasis model assessment for insulin resistance (HOMA‐IR), LDL cholesterol, and postprandial glycemia and insulin levels; however, it exclusively examined yacon syrup. To our knowledge, there has been no meta‐analysis of the previously reported effects of yacon on glycemic control and lipid profiles. Therefore, the present study performed a systematic review and meta‐analysis of all published clinical trials to provide a more precise estimation of the effects of yacon on glycemic control and lipid profiles.

## 2 Methods

In conducting this meta‐analysis and review study, which sought to examine the effects of yacon consumption on glycemic control and lipid profiles, the research adhered to the guidelines established by the Preferred Reporting Items for Systematic Reviews and Meta‐Analyses (PRISMA) [38]. This study's registration number in PROSPERO is CRD420251028504.

### 2.1 Data Source and Electronic Search

A systematic search was performed across multiple electronic databases, including ISI Web of Science, Cochrane Library, PubMed, and Scopus, to explore articles pertinent to the study title. This search was conducted until June 4, 2024, without restrictions on time or language, aiming to identify research investigating the impact of yacon consumption on glycemic control and lipid profiles. To conduct this systematic search, the following keywords and terms were employed: Yacon, Smallanthus sonchifolius, intervention, RCTs, and trials (Table S1). To achieve a thorough coverage of relevant literature and to mitigate the risk of overlooking articles not captured in systematic searches, we undertook a manual search of the initial pages of Google Scholar. Furthermore, we meticulously examined the reference lists of pertinent articles related to this topic.

### 2.2 Study Selection and Inclusion/Exclusion Criteria

To streamline the review process and identify pertinent studies, we established the following inclusion criteria: participants must be aged 18 years and older, the studies must be RCTs, and they should provide information on daily yacon consumption, including the daily dosage, frequency of use, and duration of the intervention (more than 2 weeks). To accomplish this objective, the PICOS criteria (Patients, Intervention, Comparator, Outcome, Study design) were employed. The criteria were as follows: (a) Participants included adults aged 18 years and older; (b) the studies utilized yacon as an active ingredient in either syrup or powder form; (c) a placebo comparison group was incorporated; (d) the studies evaluated glycemic and lipid profiles; and (e) the studies were designed as RCTs. The criteria for exclusion in this study included several categories: unpublished articles, conference papers, letters to the editor, animal studies, and review articles. Moreover, studies lacking a control group, those involving pregnant or lactating individuals, were excluded from consideration. Studies that included co‐interventions such as combined dietary regimens, exercise programs, or other lifestyle modifications were also excluded if their design did not permit the isolated evaluation of yacon's effects. However, we did include trials where the only distinction between the intervention and control groups was the addition of yacon (e.g., yacon + standard diet compared to standard diet alone, or yacon + medication versus medication alone). These designs provided a valid comparison and enabled a clear attribution of the observed outcomes to yacon supplementation.

Two reviewers independently (MN and MJ) evaluated the articles obtained from the systematic search after removing duplicates. Articles that failed to meet the inclusion criteria were excluded from the review. The remaining articles, along with those for which we could not determine inclusion or exclusion based on the title and abstract, underwent a full‐text assessment. After conducting a thorough review of the full text of the articles, the reviewers assessed them for inclusion in the final analysis according to the established criteria. Any disagreements between the two reviewers were addressed through discussions with the corresponding author (AH) of the study, ultimately leading to a well‐rounded conclusion.

### 2.3 Data Extraction and Quality Assessment

To conduct a thorough analysis of the included studies, it is crucial to extract the pertinent data from relevant articles. For this purpose, two individuals independently (MN and MJ) performed the data extraction. To assess the impact of yacon consumption on lipid profiles and glycemic control, the mean and standard deviation (SD) of each component (fasting blood sugar (FBS), insulin, HOMA‐IR, LDL, high‐density lipoprotein (HDL), TC, and TG) were obtained for both the intervention and control groups, before and after the intervention. The necessary data for reviewing and accurately describing the articles was gathered. This information included the first author's last name, the year of publication, the study's location, sample size, average age of participants, their health status, the study design, and the dose and duration of the intervention for both the intervention and control groups.

Cochrane guidelines and tools were utilised to evaluate the quality of the articles [39]. Using this framework, articles were assessed across seven domains. Based on these domains, the articles were categorised into three risk levels based on the scores assigned to each domain: high, low, and unclear risk of bias. The necessary data for this evaluation is also considered during the extraction of other information. Cohen's kappa of 0.76 between the two researchers' (MN and MJ) evaluations showed a high degree of agreement.

### 2.4 Statistical Analysis

This study utilised the mean and SD to assess the impact of yacon consumption on lipid profiles and glycemic indices. In instances where studies did not provide means and SD, we transformed alternative indicators into means and SD. This conversion was necessary to ensure that the final analysis was conducted using similar effect sizes [40]. To perform the analysis, we first calculated the mean (SD) change. For the mean change, we subtracted the mean of each outcome before the intervention from the mean after the intervention for both the intervention and control groups. Additionally, to compute the change in SD for each outcome, we utilised the following formula: [(SD_1_)^2^ + (SD_2_)^2^] − [2 × *r* × SD_1_ × SD_2_], which denote the standard deviations of the respective groups, with a correlation coefficient (*r*) assumed to be 0.6 [40]. To mitigate the influence of heterogeneity among studies on the overall results of the analysis, a random effects analysis was conducted using the Der Simonian and Laird method with bias‐corrected weighted mean differences (WMD), accompanied by 95% confidence intervals (CI) [41]. To consider the heterogeneity between studies, we utilised two approaches: the *I* ^2^ and the Cochrane *Q*‐test. We classified high heterogeneity in this study as an I‐squared value exceeding 50% and a *p*‐value from the Cochrane *Q*‐test of less than 0.05. Furthermore, to identify the sources of this heterogeneity and to enhance our understanding of the results, we performed an analysis that was pre‐specified a priori in the study protocol based on age, duration of intervention (in weeks), and gender. A sensitivity analysis was conducted to evaluate the influence of individual articles on the overall results. Begg's rank correlation test and Egger's regression asymmetry test were applied to statistically analyse publication bias. A *p*‐value of less than 0.05 was deemed significant in this study. Data extraction and analysis were performed using Stata statistical software version 14 and Microsoft Excel.

## 3 Results

### 3.1 Literature Search

**FIGURE 1:**
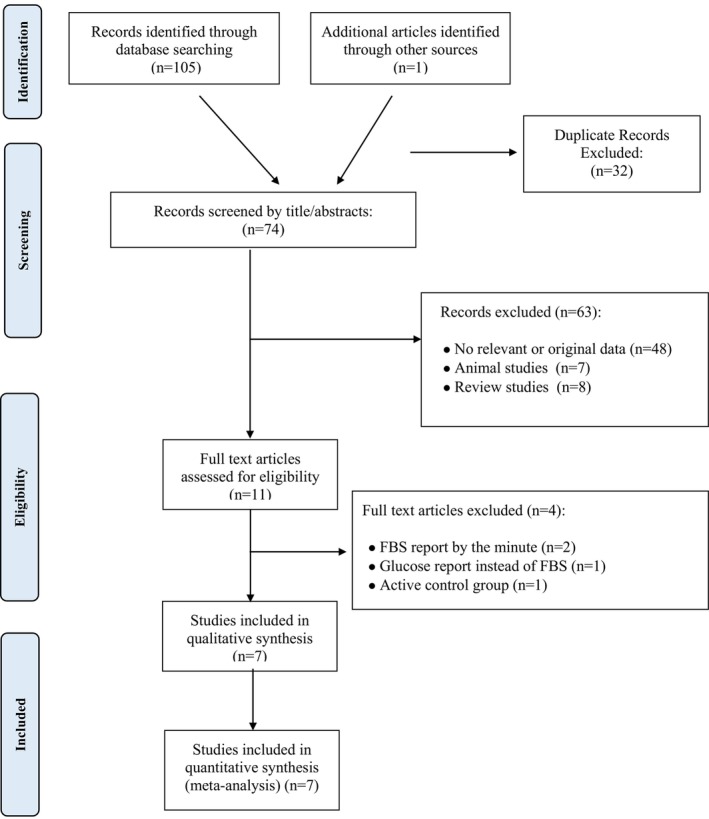
Flow chart of the number of studies identified and selected into the meta‐analysis.

Following a systematic review of the databases to identify relevant articles, a total of 104 articles were identified. An additional article was discovered through a manual search, culminating in a total of 105 studies in the initial search. Thirty‐two studies were removed due to duplication, ensuring that only one copy of each was retained. The remaining 73 articles were then screened based on their titles and abstracts. After the screening process, 63 studies were excluded for being irrelevant, animal studies, or reviews. Ten articles were selected for a more thorough evaluation to determine if they met the inclusion criteria. After reviewing the full text of these 10 articles, two studies were excluded because they had irrelevant topics [12, 42] and one study was excluded because it had an active control group [43], leaving 7 studies for the final analysis (Figure 1).

### 3.2 Results From Quality Assessment

An evaluation using the Cochrane tool to assess the quality of the included studies indicated that four studies [13, 44, 45, 46] had a high risk of bias and three articles were classified as having an unclear risk [34, 47, 48]. Further details regarding the quality of these articles are demonstrated in (Table 1).

**TABLE 1:** Risk of bias for randomised controlled trials, assessed according to the Revised Cochrane risk‐of‐bias tool for randomised trials.

Publications	Random sequence generation	Allocation concealment	Selective reporting	Blinding (participants and personnel)	Blinding (outcome assessment)	Incomplete outcome data	Other source of bias
1. Arshraf (2022) [44]	L	U	L	H	H	L	L
2. Dimarucot (2015) [45]	L	U	L	H	H	L	L
3. Dionísio (2020) [47]	L	L	L	L	U	L	L
4. Genta (2009) [13]	L	U	L	L	U	H	L
5. Scheid (2014) [34]	L	L	L	L	U	L	L
6. Ribeiro (2021) [48]	L	L	L	L	U	L	L
7. Cabral (2024) [46]	L	L	L	L	L	H	L

Abbreviations: H, high risk of bias; L, low risk of bias; U, unknown.

### 3.3 Characteristics of the Included Studies

A total of seven studies were included in the final analysis, with two specifically focusing on women [13, 46]. These studies were conducted between 2009 and 2024. The cumulative participant count for the analysis was 239, with an average age of 45.36 years; however, one study did not report the mean age [44]. The interventions lasted between 2 and 14 weeks and involved either yacon powder or syrup. Among the studies, four were conducted on healthy individuals [34, 46, 47, 48], two focused on people with dyslipidaemia [13, 45] and one involved elderly participants [44]. Additionally, four studies were conducted in Brazil [34, 46, 47, 48], while one study took place in each of the Philippines [45], Argentina [13], and Pakistan [44] (Table 2).

**TABLE 2:** Demographic characteristics of the included studies.

First author (year)	Location	Study design	Health status	Sex	Sample size	Duration (week)	Mean age (year)	Baseline BMI (kg/m^2^)	Intervention group	Comparator group	Outcome
1. Arshraf (2022) [44]	Pakistan	RCT	Elderly subjects	Both	20	8	NA	NA	20 g of yacon powder twice a day	Usual diet	FBS
2. Dimarucot (2015) [45]	Philippines	RCT	Dyslipidemia	Both	19	6	54.07	24.37	0.14 g/KBW/day of yacon + Statin	Statin	TC/TG/LDL/HDL
3. Dionísio (2020) [47]	Brazil	RCT	Healthy	Both	30	2	40.9	25.1	40 g yacon syrup	Placebo	FBS/Insulin/TC/TG/LDL/HDL
4. Genta (2009) [13]	Argentina	RCT	Slightly dyslipidemic pre‐menopausal	Female	55	14	40.5	33.5	0.14 g fructooligosaccharides/kg body weight/day	Placebo	FBS/Insulin/HOMA/TC/TG/LDL/HDL
5. Scheid (2014) [34]	Brazil	RCT	Healthy	Both	74	9	67.11	27.89	Freeze‐dried powdered yacon	Placebo	FBS/Insulin/HOMA/TC/TG/LDL/HDL
6. Ribeiro (2021) [48]	Brazil	RCT	Healthy	Both	26	6	31.3	30.4	25 g of yacon flour + breakfast drink (350 mL)	Breakfast drink (350 mL)	FBS/Insulin/HOMA/TC/TG/LDL/HDL
7. Cabral (2024) [46]	Brazil	RCT	Healthy	Female	15	4	38.3	33.74	12 mL of yacon syrup	12 mL of maltodextrin syrup (placebo)	FBS/TC/TG/LDL/HDL

Abbreviations: BMI, body mass index; C, total cholesterol; FBS, fasting blood sugar; HDL, high‐density lipoprotein; LDL, low‐density lipoprotein; NA, not available; RCT, randomised controlled trial; TG, triglycerides.

### 3.4 Findings From Meta‐Analysis

#### 3.4.1 Efect of Yacon on Glycemic Control

**FIGURE 2:**
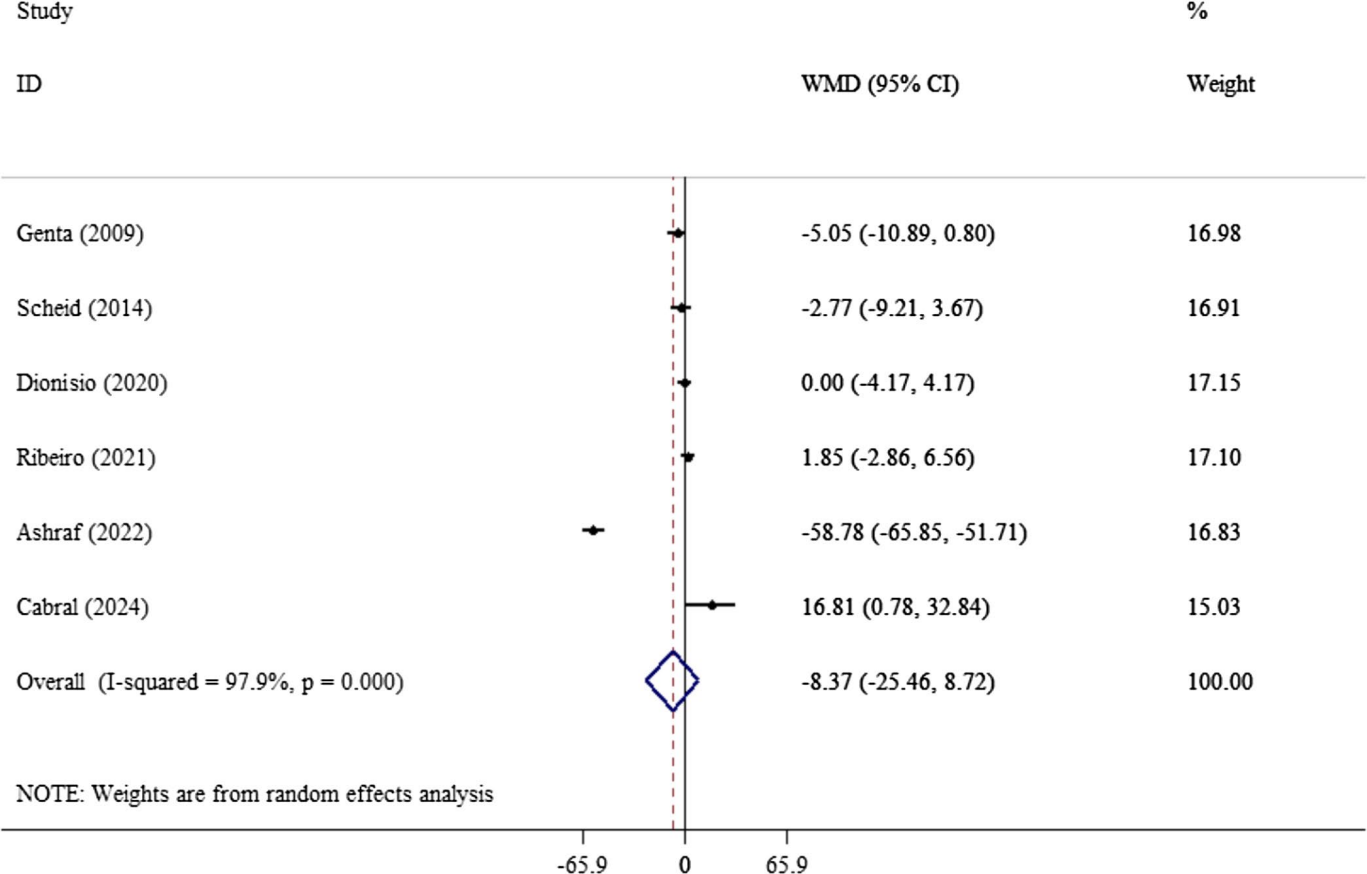
Forest plot of the effect of yacon supplementation on FBS. Analyses were performed using a random‐effects model (DerSimonian and Laird). Results are expressed as WMD with 95% CI. Heterogeneity was evaluated by *I* ^2^ statistic.

**FIGURE 3:**
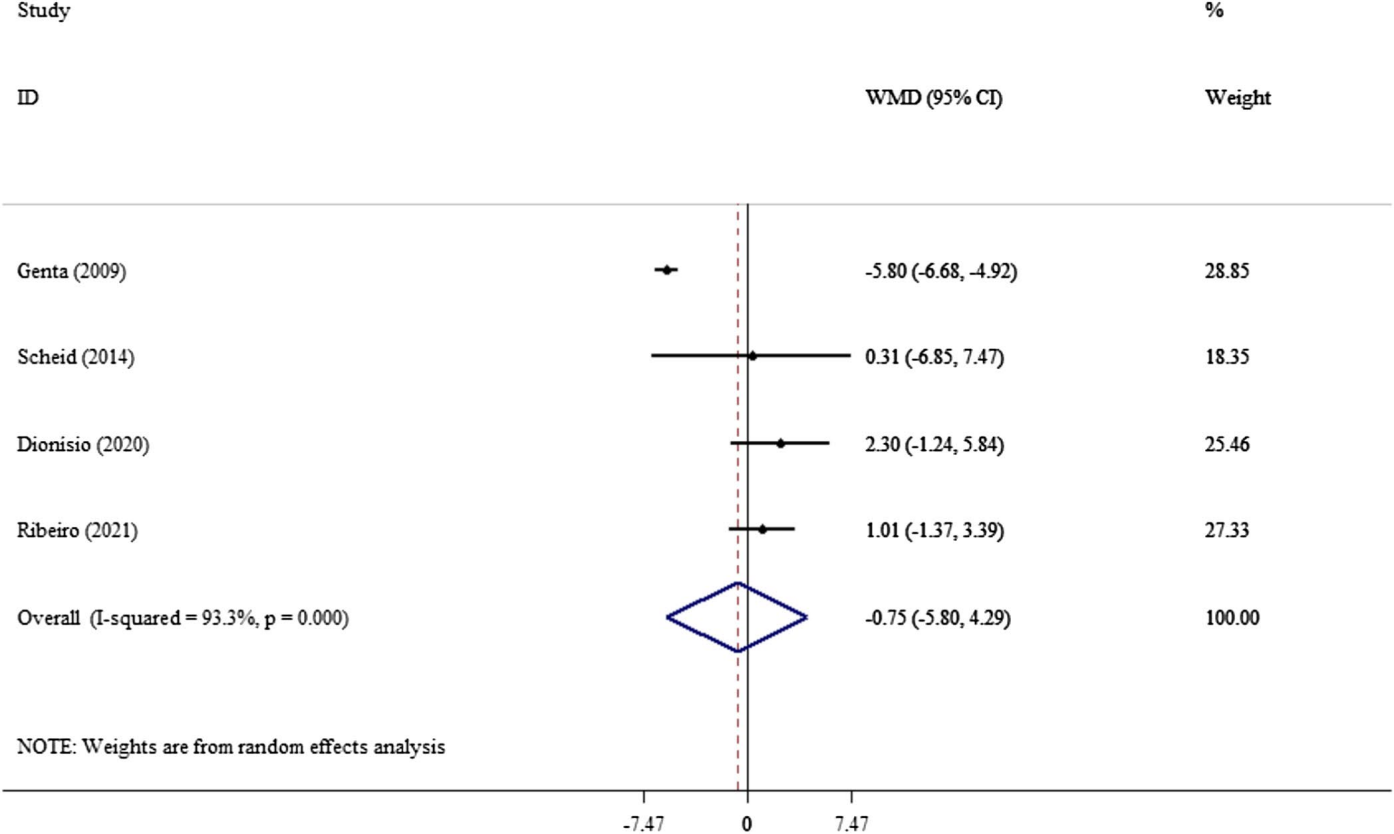
Forest plot of the effect of yacon supplementation on insulin. Analyses were performed using a random‐effects model (DerSimonian and Laird). Results are expressed as WMD with 95% CI. Heterogeneity was evaluated by *I* ^2^ statistic.

**FIGURE 4:**
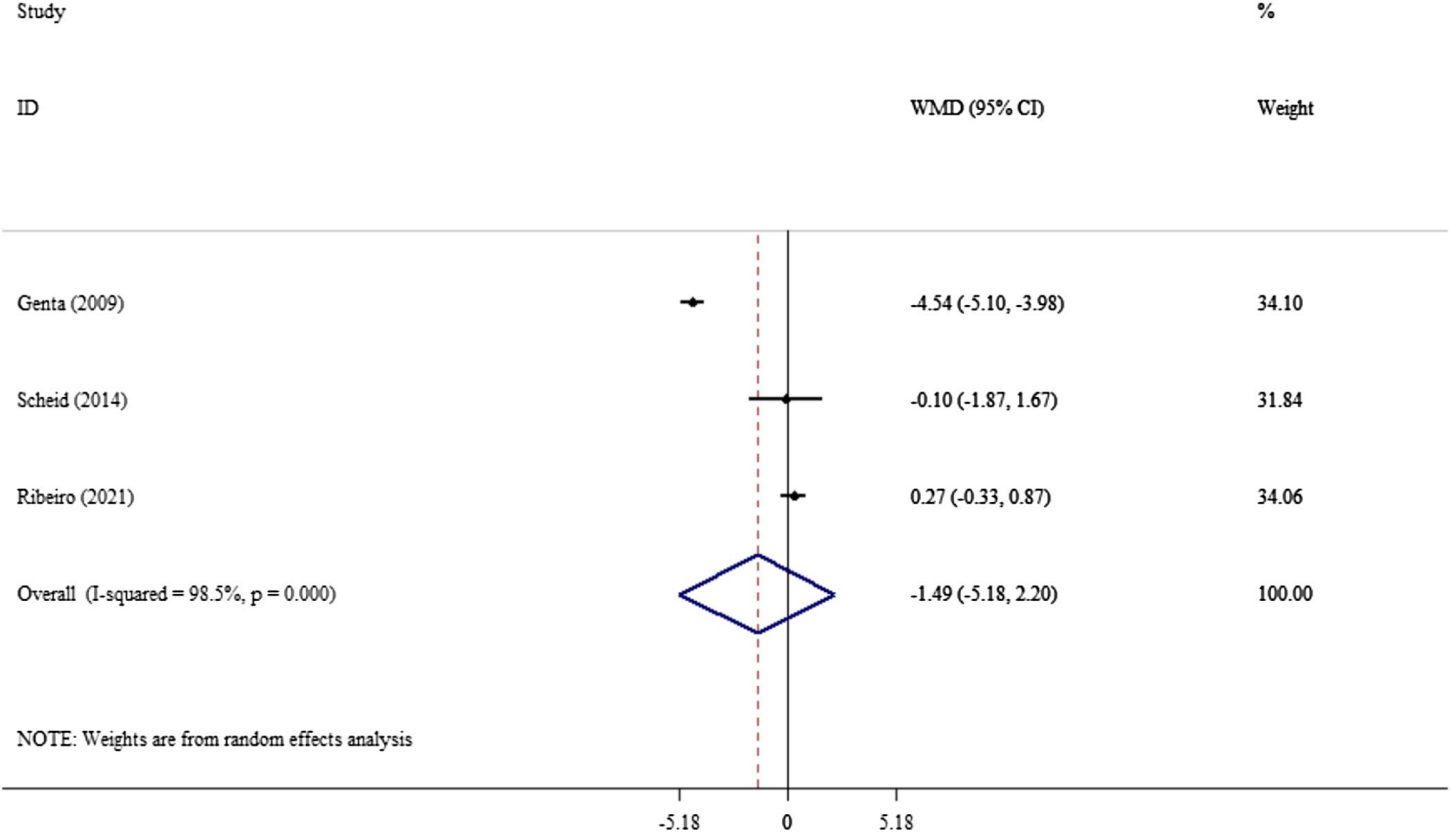
Forest plot of the effect of yacon supplementation on HOMA. Analyses were performed using a random‐effects model (DerSimonian and Laird). Results are expressed as WMD with 95% CI. Heterogeneity was evaluated by the *I* ^2^ statistic.

The final analysis included seven studies that investigated the effects of yacon on glycemic control and lipid profiles. The analysis of six, three, and four studies, respectively, revealed that yacon consumption had no significant impact on serum FBS levels (WMD: −8.37 mg/dL; 95% CI: −25.46, 8.72, *p* = 0.33; *I* ^2^: 97.9%) (Figure 2), insulin levels (WMD: −0.75 pmol/L; 95% CI: −5.80, 4.29, *p* = 0.76; *I* ^2^: 93.3%) (Figure 3), and HOMA‐IR (WMD: −1.49 unit; 95% CI: −5.18, 2.20, *p* = 0.42; *I* ^2^: 98.5%) (Figure 4). To explore the sources of heterogeneity among studies on FBS, we performed a subgroup analysis based on age, gender, and the duration of the intervention (Table 3). However, we did not conduct subgroup analyses for insulin and HOMA‐IR due to the limited number of studies available. Our findings indicated that yacon consumption significantly reduced FBS during intervention periods exceeding 8 weeks (WMD: −18.9 mg/dL; 95% CI: −22.6, −15.2, *p* < 0.001). This reduction was observed across both sexes (WMD: −7.90 mg/dL; 95% CI: −10.5, −5.28, *p* < 0.001) and was particularly pronounced in individuals over the age of 40 (WMD: −10.6 mg/dL; 95% CI: −13.3, 7.85, *p* < 0.001) (Table 3).

**TABLE 3:** Subgroup analysis of included randomised controlled trials in a meta‐analysis of the effect of yacon consumption on FBS and lipid profiles.

Group	No. of trials	WMD (95% CI)	*p*	*I* ^*2*^ (%)	P‐heterogeneity	*P* for between subgroup heterogeneity
FBS						
Duration (week)						
< 8	3	1.40 (−1.67, 4.46)	0.37	50.2	0.13	< 0.001
≥ 8	3	−18.9 (−22.6, −15.2)	< 0.001	98.8	< 0.001	
Sex						
Both	4	−7.90 (−10.5, −5.28)	< 0.001	98.7	< 0.001	0.08
Female	2	−2.48 (−7.97, 3.01)	0.37	84.1	0.01	
Age						
< 40	2	3.04 (−1.48, 7.55)	0.18	67.5	0.07	< 0.001
≥ 40	4	−10.6 (−13.3, −7.85)	< 0.001	98.6	< 0.001	
TC						
Duration (week)						
< 8	4	−6.18 (−13.6, 1.32)	0.10	27.9	0.24	0.39
≥ 8	2	−0.13 (−11.9, 11.6)	0.98	0.0	0.92	
Sex						
Both	4	−2.91 (−11.7, 5.95)	0.51	28.5	0.24	0.63
Female	2	−6.00 (−15.0, 3.02)	0.19	0.0	0.49	
Age						
< 40	2	−6.93 (−16.4, 2.56)	0.15	0.0	0.72	0.49
≥ 40	4	−2.44 (−10.9, 6.04)	0.57	30.1	0.23	
TG						
Duration (week)						
< 8	4	−0.30 (−13.3, 12.7)	0.96	0.0	0.84	0.65
≥ 8	2	4.42 (−11.4, 2.02)	0.58	0.0	0.70	
Sex						
Both	4	−0.28 (−12.7, 12.1)	0.96	0.0	0.84	0.61
Female	2	5.20 (−11.9, 22.3)	0.55	0.0	0.75	
Age						
< 40	2	−5.20 (−28.6, 18.2)	0.66	0.0	0.73	0.52
≥ 40	4	3.14 (−8.00, 14.2)	0.58	0.0	0.87	
LDL						
Duration (week)						
< 8	4	−3.42 (−10.0, 3.22)	0.31	66.8	0.02	0.03
≥ 8	2	−15.6 (−24.9, −6.47)	0.001	89.0	0.003	
Sex						
Both	4	−2.67 (−9.47, 4.12)	0.44	66.4	0.03	0.01
Female	2	−16.0 (−24.9, −7.18)	< 0.001	87.8	0.004	
Age						
< 40	4	−3.41 (−11.6, 4.85)	0.41	0.0	0.68	0.18
≥ 40	2	−10.7 (−17.8, −3.62)	0.003	85.5	< 0.001	
HDL						
Duration (week)						
< 8	4	1.12 (−1.30, 3.54)	0.36	67.2	0.02	0.71
≥ 8	2	1.93 (−1.73, 5.60)	0.30	0.0	0.33	
Sex						
Both	4	0.88 (−7.11, 8.88)	0.16	58.5	0.06	0.63
Female	2	14.0 (−1.86, 29.9)	0.84	64.1	0.09	
Age						
< 40	4	−1.45 (−4.66, 1.75)	0.37	0.0	0.57	0.02
≥ 40	2	3.21 (0.61, 5.80)	0.01	40.2	0.17	

Abbreviations: FBS, fasting blood sugar; HDL, high‐density lipoprotein; LDL, low‐density lipoprotein; TC, total cholesterol; TG, triglycerides; WMD, weight mean difference.

Sensitivity analysis was also performed for all three outcomes separately, which were not statistically significant in any of the cases. The results of Egger's regression asymmetry test showed *p*‐values of 0.67, 0.15, and 0.81 for FBS, Insulin, and HOMA‐IR, respectively, indicating statistical insignificance. Similarly, Begg's rank correlation tests produced *p*‐values of 0.45, 1.00, and 1.00 for the same measures, confirming the absence of significant publication bias.

#### 3.4.2 Efect of Yacon on Lipid Profiles

**FIGURE 5:**
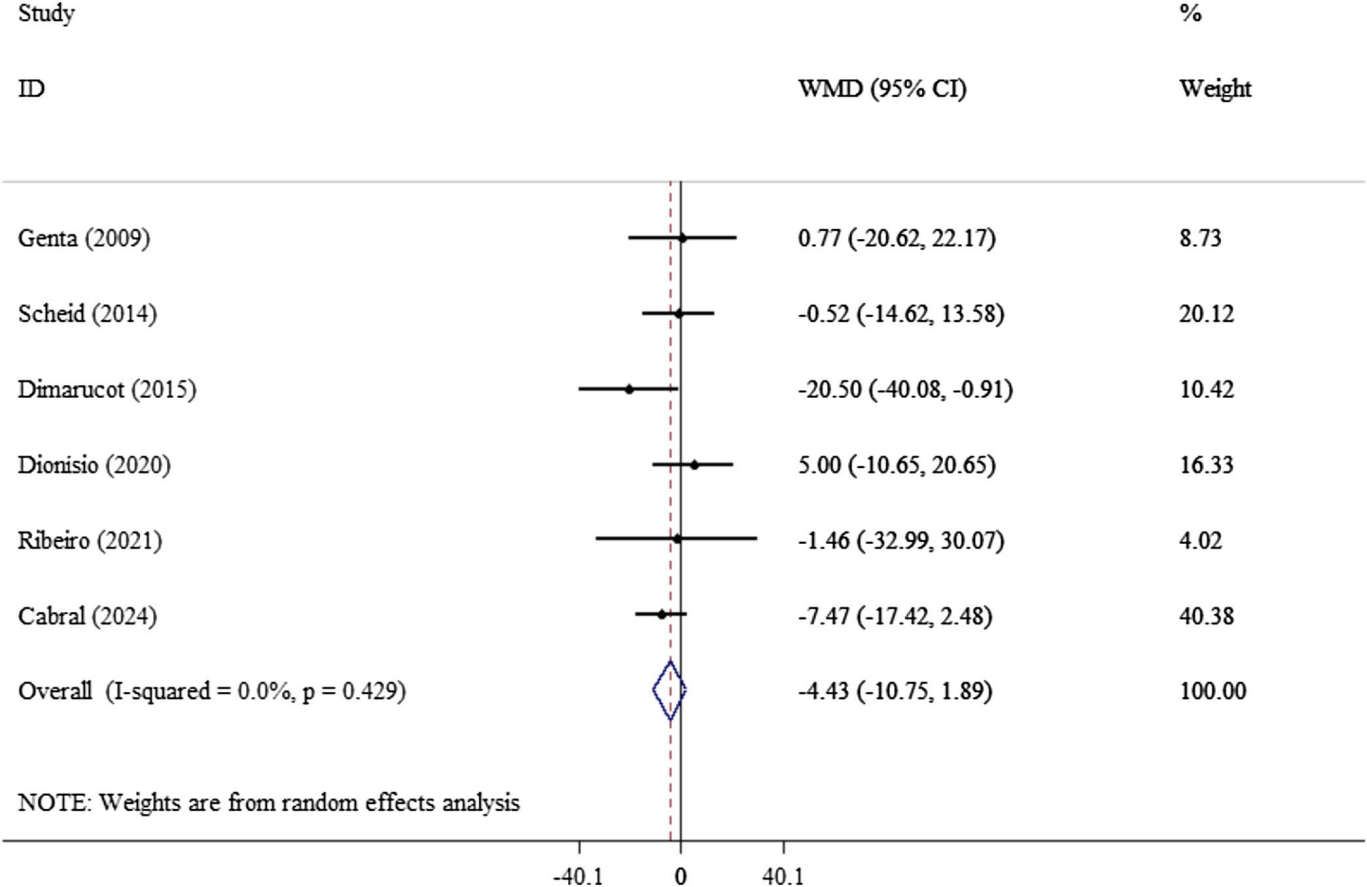
Forest plot of the effect of yacon supplementation on TC. Analyses were performed using a random‐effects model (DerSimonian and Laird). Results are expressed as WMD with a 95% CI. Heterogeneity was evaluated by the *I* ^2^ statistic.

**FIGURE 6:**
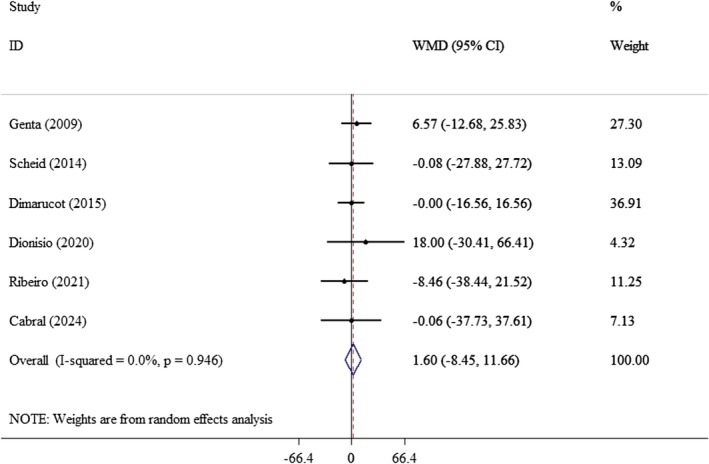
Forest plot of the effect of yacon supplementation on TG. Analyses were performed using a random‐effects model (DerSimonian and Laird). Results are expressed as WMD with 95% CI. Heterogeneity was evaluated by the *I* ^2^ statistic.

**FIGURE 7:**
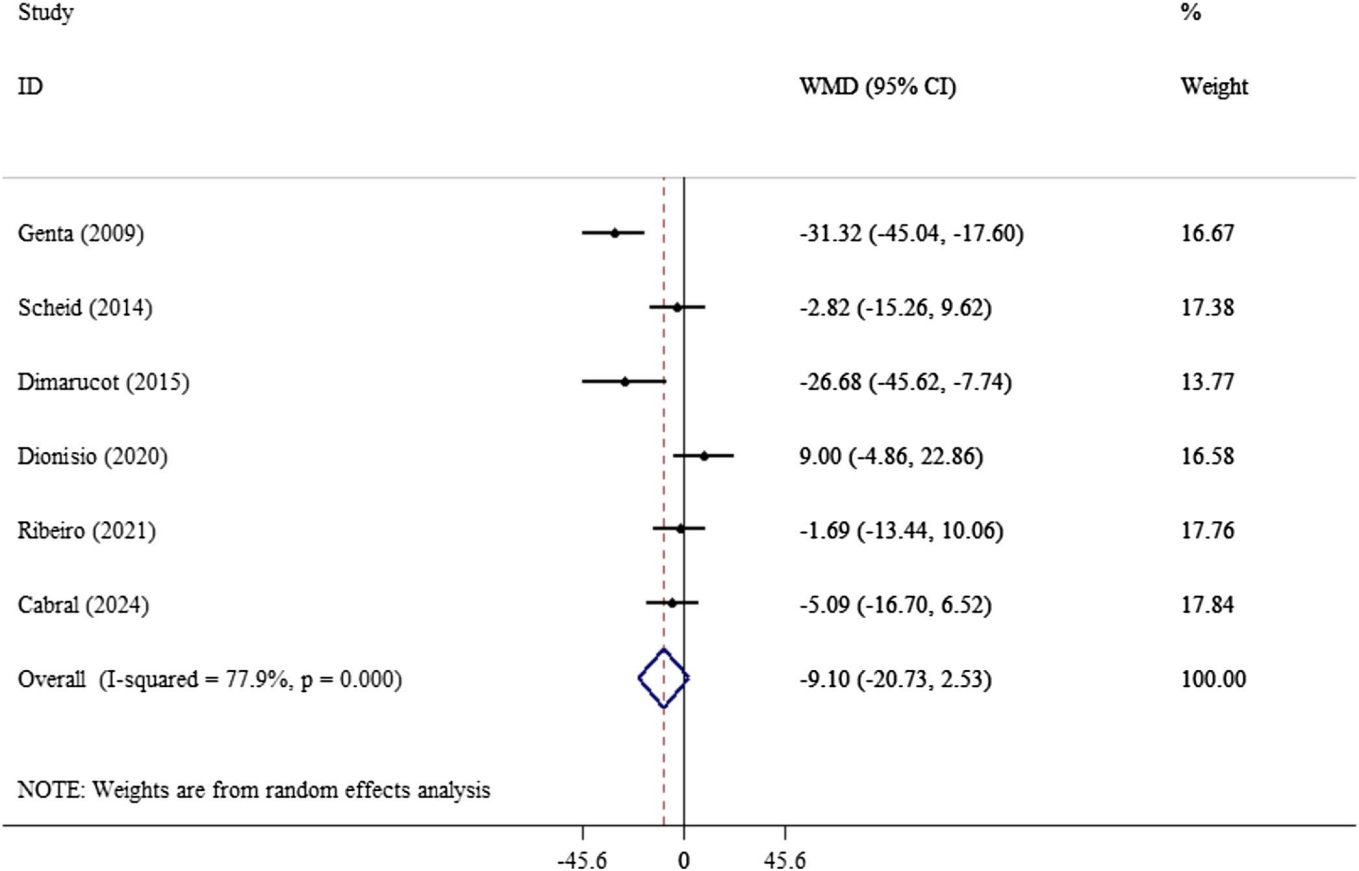
Forest plot of the effect of yacon supplementation on LDL. Analyses were performed using a random‐effects model (DerSimonian and Laird). Results are expressed as WMD with 95% CI. Heterogeneity was evaluated by *I* ^2^ statistic.

**FIGURE 8:**
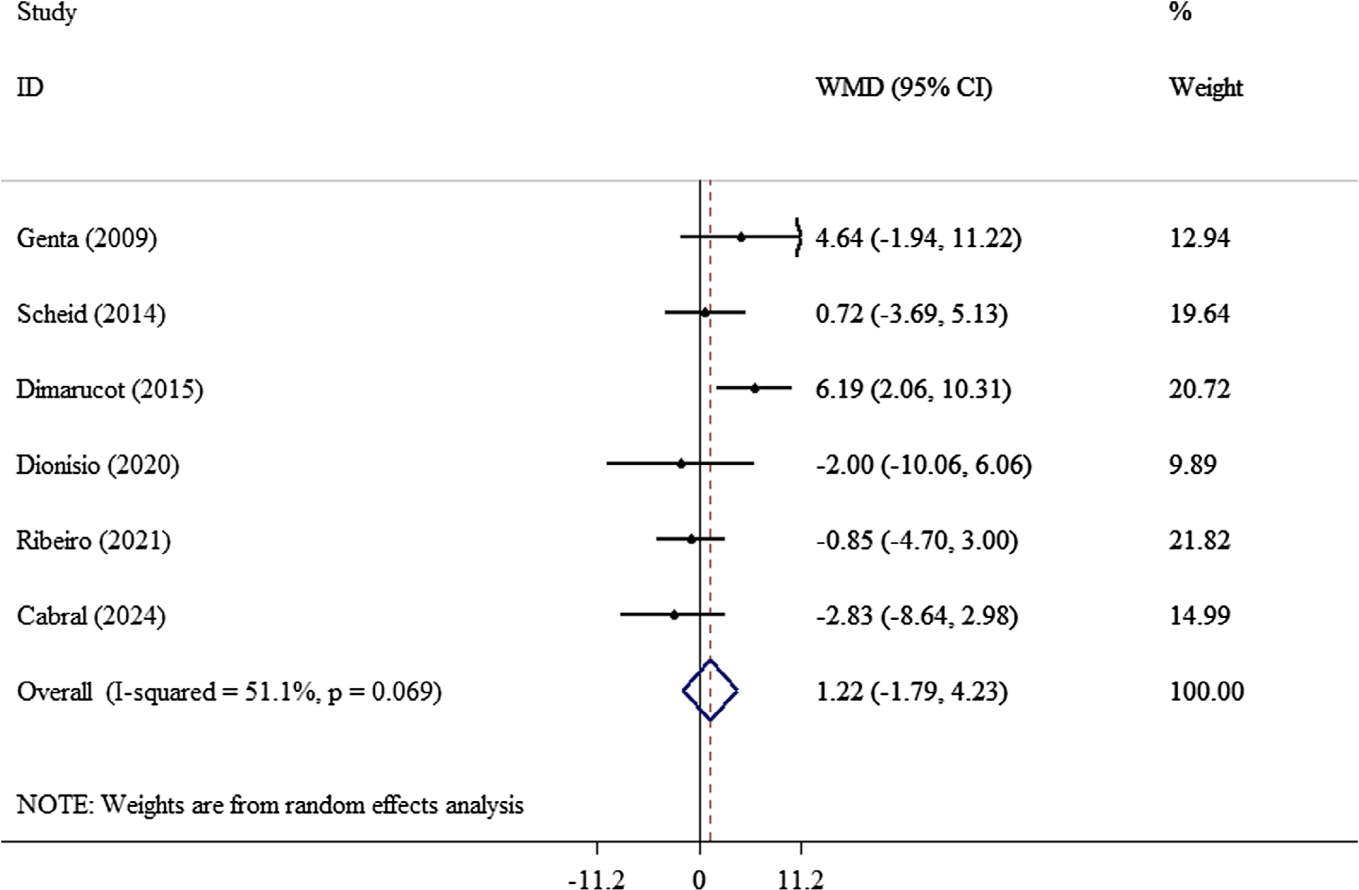
Forest plot of the effect of yacon supplementation on HDL. Analyses were performed using a random‐effects model (DerSimonian and Laird). Results are expressed as WMD with 95% CI. Heterogeneity was evaluated by *I* ^2^ statistic.

An analysis of six studies investigating the effects of yacon on lipid profiles indicated that yacon consumption did not have a significant impact on TC (WMD: −4.43 mg/dL; 95% CI: −10.75, 1.89, *p* = 0.17; *I* ^2^: 0.0%) (Figure 5). Similarly, there was no significant effect on TG (WMD: 1.60 mg/dL; 95% CI: −8.45, 11.66, *p* = 0.75; *I* ^2^: 0.0%) (Figure 6), LDL (WMD: −9.10 mg/dL; 95% CI: −20.73, 2.53, *p* = 0.12; *I* ^2^: 77.9%) (Figure 7) and HDL (WMD: 1.22 mg/dL; 95% CI: −1.79, 4.23, *p* = 0.42; *I* ^2^: 51.1%) (Figure 8). To enhance the clarity of the findings and to identify potential sources of heterogeneity among the studies, subgroup analyses were conducted based on variables such as age, gender, and duration of intervention to lipid profile outcomes (Table 3). A subgroup analysis revealed that consuming yacon for more than 8 weeks significantly reduced LDL levels (WMD: −15.6 mg/dL; 95% CI: −24.9, −6.47, *p*‐value: 0.001). This reduction was particularly notable in women (WMD: −16.0 mg/dL; 95% CI: −24.9, −7.18, *p* < 0.001). Additionally, a significant decrease in LDL was observed in individuals over the age of 40 (WMD: −10.7 mg/dL; 95% CI: −17.8, −3.62, *p*‐value: 0.003). In this same group, yacon consumption also led to a significant increase in HDL levels (WMD: 3.21 mg/dL; 95% CI: 0.61, 5.80, *p*‐value: 0.01). However, no significant association was found with other outcomes (Table 3).

Sensitivity analysis indicated that none of the items were statistically significant, suggesting that the overall results remained unaffected by any individual article. The P‐values from Begg's rank correlation tests for TG, TC, LDL, and HDL were 1.00, 0.45, 1.00, and 1.00, respectively. Furthermore, the P‐values from Egger's regression asymmetry test for these same outcomes were 0.85, 0.81, 0.33, and 0.70, respectively, none of which reached statistical significance.

The outcomes remained unchanged when *r* = 0.3 and *r* = 0.9 were used as correlation coefficients (Table S2).

## 4 Discussion

This meta‐analysis is the first to aggregate data on the effects of yacon consumption on glycemic control and lipid profiles in adults. The findings indicate that yacon supplementation had no significant impact on FBS, TC, insulin levels, HOMA‐IR, TG, LDL‐cholesterol, and HDL‐cholesterol. However, based on subgroup analyses, yacon supplementation improved FBS over 8 weeks, particularly in both genders and individuals over 40, suggesting potential benefits for glycemic management in this at‐risk demographic. Additionally, yacon reduced LDL‐cholesterol levels in women and older adults and increased HDL‐cholesterol in those aged 40 and above, indicating favourable shifts in lipid profiles. These findings highlight yacon's potential role in improving metabolic health, particularly for older adults, and emphasise the need for personalised dietary recommendations and further research into its clinical applications.

Yacon (*Smallanthus sonchifolius*), a perennial herb from the Asteraceae family, is native to the Andean regions of South America and is also grown in other countries, such as Brazil [27]. This plant stores carbohydrates as β‐1,2‐FOS, which can withstand hydrolysis by enzymes in the upper gastrointestinal tract [49]. FOS specifically enhance the growth of bifidobacteria, a gram‐positive microorganism that plays a crucial role in the colon by suppressing the growth of harmful bacteria [50]. Therefore, FOS is regarded as prebiotics, defined as “a substrate that is selectively utilized by host microorganisms conferring a health benefit” [51].

Yacon has a number of health advantages, including the capacity to control blood sugar levels, enhance lipid metabolism, aid weight reduction, improve liver health, and improve digestive health [34]. Consistent with our findings, a recent study indicated that yacon supplementation did not result in significant changes in serum lipid levels, including cholesterol and triglycerides, in healthy volunteers following a two‐week intervention [47]. Additionally, research by Gomes da Silva et al., similarly found that yacon had minimal to no effect on lipid metabolism within their study population [52]. In contrast to our findings, research involving male Wistar rats demonstrated that yacon supplementation led to a significant reduction in plasma cholesterol and TG levels. Specifically, this study reported a 50.35% decrease in blood plasma TG and a 24.46% reduction in cholesterol levels due to an aqueous extract of yacon leaves, along with a 29.29% increase in HDL levels after a 14‐day treatment period [53]. Additionally, another animal study investigating the effects of yacon flour on lipid profile suggested that yacon could positively improve lipid profile over time [54]. Genta et al. conducted a study to assess the effects of yacon syrup supplementation in pre‐menopausal women who were obese and slightly dyslipidemic. The results revealed that consuming yacon syrup daily for 120 days led to a notable reduction in LDL cholesterol and a moderate rise in HDL cholesterol. However, there were no significant changes in TC or TG levels [13].

The anti‐dyslipidemic effect of yacon is attributed to its high content of FOS. These short‐chain carbohydrates are resistant to degradation and absorption in the small intestine, allowing them to reach the large intestine intact, where they act as effective prebiotics. Prebiotics are defined as “indigestible fermented food substrates that selectively promote the growth, composition, and activity of beneficial microflora in the gastrointestinal tract” [55]. Specifically, the fermentation of FOS by intestinal bacteria encourages the proliferation of beneficial lactose‐fermenting bacteria, such as Lactobacillus [56]. Several mechanisms have been suggested to explain how Lactobacillus strains and other lactic acid bacteria (LAB) reduce cholesterol levels. Beyond their direct cholesterol‐lowering effects—such as binding cholesterol in the small intestine, incorporating it into their cell membranes, and co‐precipitating it with deconjugated bile salts—these bacteria also produce bile salt hydrolase (BSH). This enzyme catalyses the deconjugation of bile salts [56], and Lactobacillus strains are particularly effective at this. In an in vivo study, these cultures were able to deconjugate 60% to 90% of bile salts [57], highlighting their potential impact on cholesterol metabolism and lipid profile enhancement. The deconjugation of bile salts results in the release of free taurine and glycine, which are reabsorbed, and free cholic acid, which is excreted in faeces [56]. This increased excretion of cholic acid reduces the hepatic pool of bile acids, leading to the downregulation of cholesterol‐7‐α‐hydroxylase (CYP7A1). This enzyme catalyses the initial rate‐limiting step in the synthesis of bile acids from cholesterol. When CYP7A1 is activated, it promotes the use of serum and hepatic cholesterol for bile acid production [58]. Consequently, decreased hepatic cholesterol levels enhance the expression of LDL receptors in hepatocytes [58]. These processes likely explain the reduction in LDL and total cholesterol associated with increased bile acid secretion. Conversely, the mechanism by which interrupting the enterohepatic circulation of bile increases HDL levels is still being explored. The farnesoid‐X receptor (FXR), a member of the nuclear receptor superfamily found in hepatocytes, is activated by primary bile acids. When activated, FXR represses the expression of apoprotein A1 (ApoA1), a major component of HDL, as well as the gene for apoprotein M (ApoM), which is important for HDL maturation [59]. Therefore, decreased activation of FXR due to lower hepatic bile acid levels may lead to increased expression of the ApoA1 and ApoM genes, potentially explaining the rise in HDL observed with increased bile excretion. Numerous studies have indicated that enhanced faecal bile excretion correlates with lower total and LDL cholesterol levels while increasing HDL cholesterol levels [58]. This aligns with other prebiotic meta‐analyses, including those focused on inulin, which have demonstrated significant improvements in lipid profiles, especially in individuals with high cholesterol. Inulin, similar to FOS, has shown promise in lowering TC and LDL cholesterol levels [60].

In terms of glycaemic control, our results align with a study involving adults with type 2 diabetes, which found that daily consumption of yacon syrup over 12 weeks did not result in significant changes in fasting blood glucose or HbA1c levels compared to control groups [61]. Similarly, an animal study with diabetic rats indicated that administration of yacon flour did not lead to significant changes in plasma glucose levels [54]. Conversely, a randomised, crossover, double‐blind clinical trial involving 40 women demonstrated that consuming yacon syrup significantly lowered postprandial glucose and insulin levels compared to a placebo. Blood samples taken at different intervals revealed reduced glucose levels at 30 min and lower insulin levels at 15, 30, and 45 min following yacon syrup intake [12]. The exact mechanisms through which the plant lowers blood glucose levels are not completely understood. However, they may involve several factors, including enhanced insulin secretion due to stimulation of pancreatic beta cells, resistance to hormones that elevate glucose release, an increase in both the quantity and sensitivity of insulin receptors, decreased glycogen breakdown, improved glucose uptake by various tissues and organs, and reduced intestinal absorption of glucose, among other possibilities [62]. Lack of significant effects of yacon on glycemic control and lipid profile can be based on several points including study design, individual variability, dosage and formulation, baseline control, duration of intervention, complex mechanisms, and lifestyle factors. This is the first study that offers several advantages that improve its contribution to understanding the effects of yacon. It serves as a comprehensive systematic review and meta‐analysis that includes all RCTs related to yacon, providing a wide‐ranging overview of the existing evidence. By not restricting the publication date or language, the study allows for a more inclusive examination of available research. The results of Egger's test suggested that publication bias is unlikely to have substantially influenced our overall findings. The lack of significant bias suggests that our results are more likely to reflect true effects rather than being skewed by unpublished or selectively reported studies. A notable strength is its standardised methodology, which enhances the study's reliability. The standardised methodology employed throughout the research allows for a consistent approach to data collection and analysis, which is crucial for minimising variability and ensuring that findings are comparable across different studies. This not only strengthens the reliability of the results but also facilitates replication in future research.

In our analysis, we employed the Cochrane Risk of Bias tool to assess the potential biases across included studies. This assessment revealed several domains with high or unclear risk, which significantly diminishes confidence in our findings. Such biases, arising from methodological flaws and a lack of transparency, could lead to either overestimation or underestimation of yacon's effects on glycaemic control and lipid metabolism. Additionally, the researchers conducted subgroup analyses to address the heterogeneity found among the included studies. The inclusion of subgroup analyses adds depth to the investigation, allowing for exploration of how yacon's effects may vary among different demographic groups such as older adults or those undertaking longer interventions. This targeted approach can highlight specific populations that may benefit most from yacon supplementation, which is valuable for tailoring dietary recommendations.

This study faces several limitations that impact the interpretation of yacon's efficacy, notably the variability in dosages used across reviewed studies, which complicates our ability to ascertain optimal dosing for glycaemic control and lipid metabolism. Additionally, high heterogeneity (*I* ^2^ > 90%) stemming from differences in participant demographics, formulation types, and study durations further complicates comparisons. The results may not be applicable to broader populations, particularly individuals with diabetes, as the sample may not represent the full spectrum of diabetes types (Type 1 and Type 2) or the various stages of the disease.

Methodological flaws identified through the Cochrane Risk of Bias tool raise concerns about the reliability of findings, while external confounding variables like dietary habits and lifestyle choices could influence individual responses to yacon. To mitigate these limitations, we implemented comprehensive inclusion criteria to capture a broad range of evidence, utilized a standardized methodology for consistency in data collection, conducted subgroup analyses to explore demographic variations, and acknowledged the influence of confounding factors to guide future research efforts in providing a clearer understanding of yacon's effects. Dosages ranging from 10 to 30 g of yacon syrup per day have been used in studies, and exceeding these amounts may increase the risk of gastrointestinal symptoms [22].

Due to all these limitations, more long‐term, well‐designed, and rigorous RCTs are needed to confirm the role of yacon as a human therapeutic strategy.

## 5 Conclusion

A comprehensive review and meta‐analysis indicated that yacon consumption in adults did not result in significant improvements in lipid profiles or glycaemic factors. However, there are indications that long‐term yacon consumption may lead to improvements in FBS and LDL cholesterol levels over time. These results suggest that while immediate effects on lipid and glycaemic profiles may not be apparent, prolonged use of yacon could offer potential benefits.

## Author Contributions

M.R.A.: data curation, formal analysis, methodology. M.N.: methodology, writing – original draft. F.S., M.M.S., M.K., S.E., F.T. and N.L.: writing – original draft. M.E.: writing – review and editing, formal analysis. M.J.: writing – original draft, methodology. A.H.: supervision, writing – review and editing.

## Consent

The authors have nothing to report.

## Conflicts of Interest

The authors declare no conflicts of interest.

## Supporting information


**Table S1:** Search syntax.
**Table S2:** Weighted mean difference and 95% confidence intervals (CIs) for the effect of yacon consumption on glycemic control and lipid profiles.

## Data Availability

The data used to support the findings of this study are available from the corresponding author upon request.
