# Supplementary material for: The Effect of Yacon Consumption on Glycemic Control and Lipid Profiles: A Systematic Review and Meta‐Analysis of Randomised Controlled Trials
Source: Endocrinol Diabetes Metab. 2025 Oct 28;8(6):e70121. doi: 10.1002/edm2.70121 (PMC12568385; doi:10.1002/edm2.70121)
Supplement: Supplementary file 1 — Table S1: Search syntax. Table S2: Weighted mean difference and 95% confidence intervals (CIs) for the effect of yacon consumption on glycemic control and lipid profiles. [file EDM2-8-e70121-s001.docx]

**Supplementary Table 1.** Search syntax.

| **Search syntax** | |
| --- | --- |
| (Yacon[tiab] OR "Smallanthus sonchifolius"[tiab]) AND (intervention[tiab] OR RCT[tiab] OR randomized[tiab] OR random[tiab] OR Randomly[tiab] OR Placebo[tiab] OR Assignment[tiab] OR trial[tiab] OR trials[tiab] OR randomised[tiab] OR "Methods"[Mesh] OR Cross-Over[tiab] OR "Double-Blind"[tiab] OR "Randomized Controlled Trial"[Publication Type] OR "Controlled Clinical Trial"[Publication Type] OR "Placebos"[Mesh] OR "Placebo Effect"[Mesh] OR "Clinical Trial"[Publication Type] OR "Clinical Trials as Topic"[Mesh] OR "Cross-Over Studies"[Mesh] OR "Double-Blind Method"[Mesh]) | **Pubmed** |
| ( TITLE-ABS-KEY ( Yacon ) OR TITLE-ABS-KEY ( "Smallanthus sonchifolius" ) AND TITLE-ABS-KEY ( intervention ) OR TITLE-ABS-KEY ( "controlled trial" ) OR TITLE-ABS-KEY ( randomized ) OR TITLE-ABS-KEY ( random ) OR TITLE-ABS-KEY ( randomly ) OR TITLE-ABS-KEY ( placebo ) OR TITLE-ABS-KEY ( assignment ) OR TITLE-ABS-KEY ( "clinical trial" ) OR TITLE-ABS-KEY ( trial ) OR TITLE-ABS-KEY ( randomised ) ) AND ( LIMIT-TO ( DOCTYPE , "ar" ) ) AND ( LIMIT-TO ( LANGUAGE , "English" ) ) AND ( LIMIT-TO ( SRCTYPE , "j" ) ) | **Scopus** |
| Yacon OR "Smallanthus sonchifolius" (All Fields) and intervention OR RCT OR randomized OR random OR Randomly OR Placebo OR Assignment OR trial OR trials OR randomized OR Cross-Over OR "Double-Blind" (All Fields) | **Web of Science** |
| (Yacon OR "Smallanthus sonchifolius"):ti,ab,kw AND (intervention OR RCT OR randomized OR random OR Randomly OR Placebo OR Assignment OR trial OR trials OR randomized OR Cross-Over OR "Double-Blind"):ti,ab,kw | **Cochrane Library** |

| **Supplementary Table 2**. Weighted mean difference and 95% confidence intervals (CIs) for the effect of yacon consumption on glycemic control and lipid profiles. | | |
| --- | --- | --- |
| **Group** | **WMD (95% CI)** | ***I^2^* (%)** |
| **Correlation coefficient (r) assumed to be 0.3** | | |
| ***FBS*** | -8.52 (-25.8, 8.78) | 96.5 |
| ***Insulin*** | -0.86 (-5.93, 4.21) | 91.4 |
| ***HOMA*** | -1.50 (-5.26, 2.25) | 98.3 |
| ***TC*** | -4.53 (-12.6, 3.57) | 0.00 |
| **TG** | 0.83 (-11.7, 13.4) | 0.00 |
| ***LDL*** | -8.75 (-20.1, 2.60) | 64.9 |
| ***HDL*** | 1.17 (-1.74, 4.08) | 24.4 |
| **Correlation coefficient (r) assumed to be 0.9** | | |
| ***FBS*** | -8.21 (-24.3, 7.93) | 99.4 |
| ***Insulin*** | -0.63 (-5.72, 4.45) | 96.4 |
| ***HOMA*** | -1.46 (-4.93, 2.00) | 98.8 |
| ***TC*** | -3.73 (-10.4, 2.97) | 65.8 |
| **TG** | 2.73 (-3.06, 8.52) | 0.00 |
| ***LDL*** | -9.40 (-21.2, 2.48) | 92.0 |
| ***HDL*** | 1.06 (-1.95, 4.08) | 82.2 |
| Abbreviations: **FBS,** Fasting blood sugar; **HOMA-IR,** Homeostasis model assessment for insulin resistance; **TC**, Total cholesterol; **TG**, Triglycerides; **LDL**, [Low-density lipoprotein](https://en.wikipedia.org/wiki/Low-density_lipoprotein); **HDL**, High-density lipoprotein; **WMD**, Weight mean difference; | | |
